# The *Ralstonia solanacearum* effector RipN suppresses plant PAMP‐triggered immunity, localizes to the endoplasmic reticulum and nucleus, and alters the NADH/NAD^+^ ratio in *Arabidopsis*

**DOI:** 10.1111/mpp.12773

**Published:** 2019-02-18

**Authors:** Yunhao Sun, Pai Li, Dong Shen, Qiaoling Wei, Jianguo He, Yongjun Lu

**Affiliations:** ^1^ School of Life Sciences Sun Yat‐sen University Guangzhou 510275 China; ^2^ State Key Laboratory of Biocontrol Sun Yat‐sen University Guangzhou 510275 China

**Keywords:** effector protein, Nudix hydrolase, plant immunity, *Ralstonia solanacearum*, RipN, type III secretion system

## Abstract

*Ralstonia solanacearum*, one of the most destructive plant bacterial pathogens, delivers an array of effector proteins via its type III secretion system for pathogenesis. However, the biochemical functions of most of these proteins remain unclear. RipN is a type III effector with unknown function(s) from the pathogen *R. solanacearum*. Here, we demonstrate that RipN is a conserved type III effector found within the *R. solanacearum* species complex that contains a putative Nudix hydrolase domain and has ADP‐ribose/NADH pyrophosphorylase activity *in vitro*. Further analysis shows that RipN localizes to the endoplasmic reticulum (ER) and nucleus in *Nicotiana tabacum* leaf cells and Arabidopsis protoplasts, and truncation of the C‐terminus of RipN results in a loss of nuclear and ER targeting. Furthermore, the expression of RipN in *Arabidopsis* suppresses callose deposition and the transcription of pathogen‐associated molecular pattern (PAMP)‐triggered immunity (PTI) marker genes under flg22 treatment, and promotes bacterial growth *in planta*. In addition, the expression of RipN in plant cells alters NADH/NAD^+^, but not GSH/GSSG, ratios, and its Nudix hydrolase activity is indispensable for such biochemical function. These results suggest that RipN acts as a Nudix hydrolase, alters the NADH/NAD^+^ ratio of the plant and contributes to *R. solanacearum* virulence by suppression of PTI of the host.

## Introduction

*Ralstonia solanacearum*, a Gram‐negative, soil‐borne, plant‐pathogenic bacterium, causes bacterial wilt in more than 200 plant species, including important crops such as potato, tomato, tobacco and banana (Genin, 2010). *Ralstonia solanacearum *is regarded as one of the most destructive pathogens worldwide because of its global geographical distribution, large heterogeneity and extreme aggressiveness (Jiang *et al.*, 2017; Mansfield *et al.*, 2012). *Ralstonia solanacearum* can live for long periods in moist soil or water, and invades the host plant vascular system through epidermal wounds created by lateral root genesis, pest bites or even agricultural tillage (Genin, 2010). Once inside the plant, *R. solanacearum* colonizes the root cortex and invades the xylem vessels, spreading to the aerial parts of the plant through the vascular system. The water transportation of the host plant is blocked by the rapid proliferation of the bacterium, resulting in the wilting and death of infected plants (Lowe‐Power *et al*., 2018).

To counter pathogen infection, plants have evolved two major defence systems to protect themselves from infection. First, pathogen‐associated molecular pattern (PAMP)‐triggered immunity (PTI) functions to recognize pathogen‐associated molecules, such as flagellin, chitin, lipopolysaccharides and peptidoglycans, to activate a rapid host defence response (Milling *et al.*, 2011; Thomma *et al.*, 2011). To overcome host‐derived PTI responses and colonize the plant, pathogens have evolved the type III secretion system (T3SS) to deliver a repertoire of effector proteins (type III effectors, T3Es) that interfere with the induced immune responses (Van Gijsegem *et al.*, 2000). To overcome the action of T3Es, plants have evolved a second immune mechanism to perceive and counter the activity of effectors through receptors—resistance (R) proteins—which, when activated, result in a sustained strong immune response referred to as effector‐triggered immunity (ETI) (Chisholm *et al.*, 2006). In total, ETI results in the induction of robust immune responses, including the hypersensitive response (HR), a form of programmed cell death, accompanied by the production of a burst of reactive oxygen species (ROS) (Poueymiro *et al.*, 2009).

Like many plant‐pathogenic bacteria, *R. solanacearum* strains infect their host by employing a T3SS to deliver effector proteins into plant cells (Cunnac *et al.*, 2004). *Ralstonia solanacearum* has been found to possess more than 70 T3Es, designated as Rips (*Ralstonia* injected proteins) (Peeters *et al.*, 2013). To date, the biological functions of several T3Es from *R. solanacearum *have been identified. For example, RipG1‐G8, which belongs to the GALA family of F‐box protein subunits, has been hypothesized to hijack the SCF (Skp1, Cullin, F‐box) E3 ubiquitin ligase complex in host cells (Angot *et al.*, 2006). Another T3E, RipP2, has been demonstrated to localize to the host nucleus, where it functions as an acetyltransferase that targets WRKY transcription factors (Tasset *et al.*, 2010), altering defence‐associated transcriptional responses. RipTPS acts as a trehalose‐6‐phosphate synthase and elicits an HR‐like response on *Nicotiana tabacum *(Poueymiro *et al.*, 2014). RipAY functions as a γ‐glutamyl cyclotransferase. Once activated by cytosolic thioredoxins of the host, it specifically degrades glutathione in plant cells to suppress plant immunity (Fujiwara *et al.*, 2016; Mukaihara *et al.*, 2016; Sang *et al.*, 2018; Wei *et al.*, 2017). AWR5 (RipA5) causes a decrease in the activity of TOR‐regulated nitrate reductase in plants to maintain normal levels of TOR and the Cdc55 homologues, which are required for *R. solanacearum* virulence (Popa *et al.*, 2016). RipAW and RipAR show E3 ubiquitin ligase activity *in vitro *and suppress PTI in plants (Nakano *et al.*, 2017). RipAK inhibits plant HR by targeting the catalase in the host peroxisome (Sun *et al.*, 2017). The functional study of T3Es from phytopathogenic bacteria has led to an increase in interest in recent years. However, the biochemical and biological functions of most *R. solanacearum* T3Es remain uncharacterized (Peeters *et al.*, 2013).

RipN was identified as a T3E from *R. solanacearum* GMI1000 by *in silico *analysis of the genome sequence, including the application of various functional genomics‐based approaches (Cunnac *et al.*, 2004); however, its biological function remains undefined (Peeters *et al.*, 2013). Alignment analysis of the protein sequence revealed that RipN contains a putative Nudix hydrolase domain in its central region. The term ‘Nudix’ refers to a conserved group of organic pyrophosphate hydrolases that hydrolyse substrates possessing the general structure of nucleoside (Nu) diphosphate (di) linked to another moiety (x), and shares a common Nudix motif (or Nudix box) of Gx5Ex5[UA]xREx2EExGU, where ‘U’ is an aliphatic hydrophobic residue (Bessman *et al*., 1996; McLennan, 2006; Yoshimura and Shigeoka, 2015).

Studies in several model organisms have demonstrated recently that Nudix proteins perform a variety of functions associated with the maintenance of cellular homeostasis through the hydrolysis of nucleoside diphosphate derivatives, including NADH (reduced form of nicotinamide adenine dinucleotide), NAD^+^ (oxidized form of nicotinamide adenine dinucleotide), ADP‐ribose (ADPr), nucleoside triphosphates (NTPs), deoxynucleoside triphosphates (dNTPs), phosphoinositol proteins and capped mRNAs (Nguyen *et al.*, 2016; Srouji *et al.*, 2017). As these substrates are regulatory molecules in plant immunity, Nudix hydrolases may play a key role in immune signalling (Fonseca and Dong, 2014; Ge *et al.*, 2007; Ogawa *et al.*, 2016). The Nudix gene family includes a large group of hydrolase proteins in plants, with 29 homologues in *Arabidopsis *(Dong and Wang, 2016). However, their roles in immunity vary, as several Nudix proteins have opposite biological functions (Fonseca and Dong, 2014; Ge *et al.*, 2007; Ogawa *et al.*, 2016). So far, Nudix effectors have been identified in plant‐pathogenic oomycetes, fungi and bacteria, suggesting that this category of effectors may play a critical virulent role in the ‘toolbox’ of plant pathogens (Dong and Wang, 2016). Recent studies have shown that PsAvr3b, a Nudix effector from *Phytophthora sojae*, has an ADPr/NADH pyrophosphorylase activity in plants and inhibits the accumulation of ROS around invasion sites (Dong *et al.*, 2011). However, apart from the conserved Nudix domain, the structures, sequences and subcellular localizations vary greatly amongst these Nudix proteins from different kingdoms (Carreras‐Puigvert *et al.*, 2017; Dong and Wang, 2016). To date, the biological roles of Nudix proteins remain poorly understood and the biological functions of bacterial Nudix effectors have not been reported (Dong and Wang, 2016).

Here, we report that the Nudix T3E RipN has a typical ADPr/NADH pyrophosphorylase activity *in vitro*, localizes to the plant endoplasmic reticulum (ER) and nucleus, affects the NADH/NAD^+^ ratio of plants and inhibits the PTI of the host.

## Results

### RipN is a conserved T3E in the *R. solanacearum* species complex and contains a Nudix hydrolase domain

**Figure 1:**
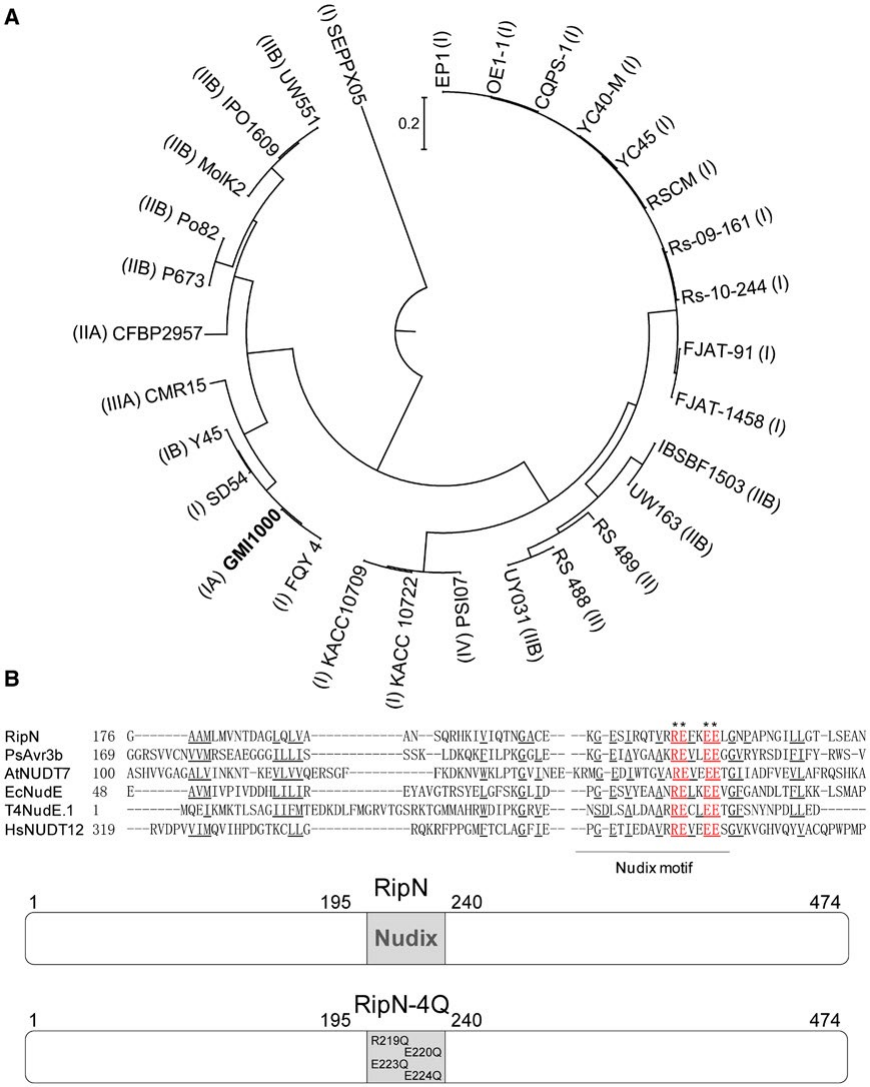
RipN is a conserved type III effector within the *Ralstonia solanacearum* species complex and contains a Nudix hydrolase motif. (A) Phylogenetic analysis of RipN from 30 *R. solanacearum *representative strains of the whole species diversity. The phylogenetic tree was constructed by MEGA5.05 software. (B) The amino acid sequences of the central region of RipN and the catalytic domain of the Nudix hydrolase family proteins were aligned using ClustalW software. Identical and highly conserved amino acid residues are shown underlined and the key amino acids of the Nudix hydrolase motif are shown in red. The catalytically inactive mutant RipN‐4Q was constructed by changing the essential amino acids at positions 219, 220, 223 and 224 to glutamine.

The *R. solanacearum *species complex exhibits a high degree of genetic diversity, and comprises different phylotypes that reflect the region of origin of the isolates. Specifically, phylotypes I, II and III originate from Asia, America and Africa, respectively, whereas phylotype IV strains originate from Indonesia, Japan and Australia (Gutarra *et al.*, 2017; Stulberg and Huang, 2015). Full genome sequences from 30 representative strains of the different phylotypes are now available. We were able to detect the presence of *ripN* in all of these analysed strains. By using this dataset, we reconstructed the RipN phylogeny in the *R. solanacearum* species complex (Fig. 1A). Phylogenetic analysis revealed that RipN is a conserved effector in the *R. solanacearum* species complex, and the diversity of RipN is closely related to the geographical distribution of the pathogen. The alignment of the protein sequence revealed that RipN contains a putative Nudix hydrolase domain in its central region (Fig. 1B). It has been reported that mutations on four hypothetical catalytic residues of PsAvr3b, which resemble the RipN positions 219, 220, 223 and 224, completely abolish the hydrolase activity of PsAvr3b (Dong *et al.*, 2011). We constructed the putative catalytically inactive mutant RipN‐4Q by mutation of these essential residues into glutamine.

### RipN has ADPr/NADH pyrophosphorylase activity *in vitro*

**Figure 2:**
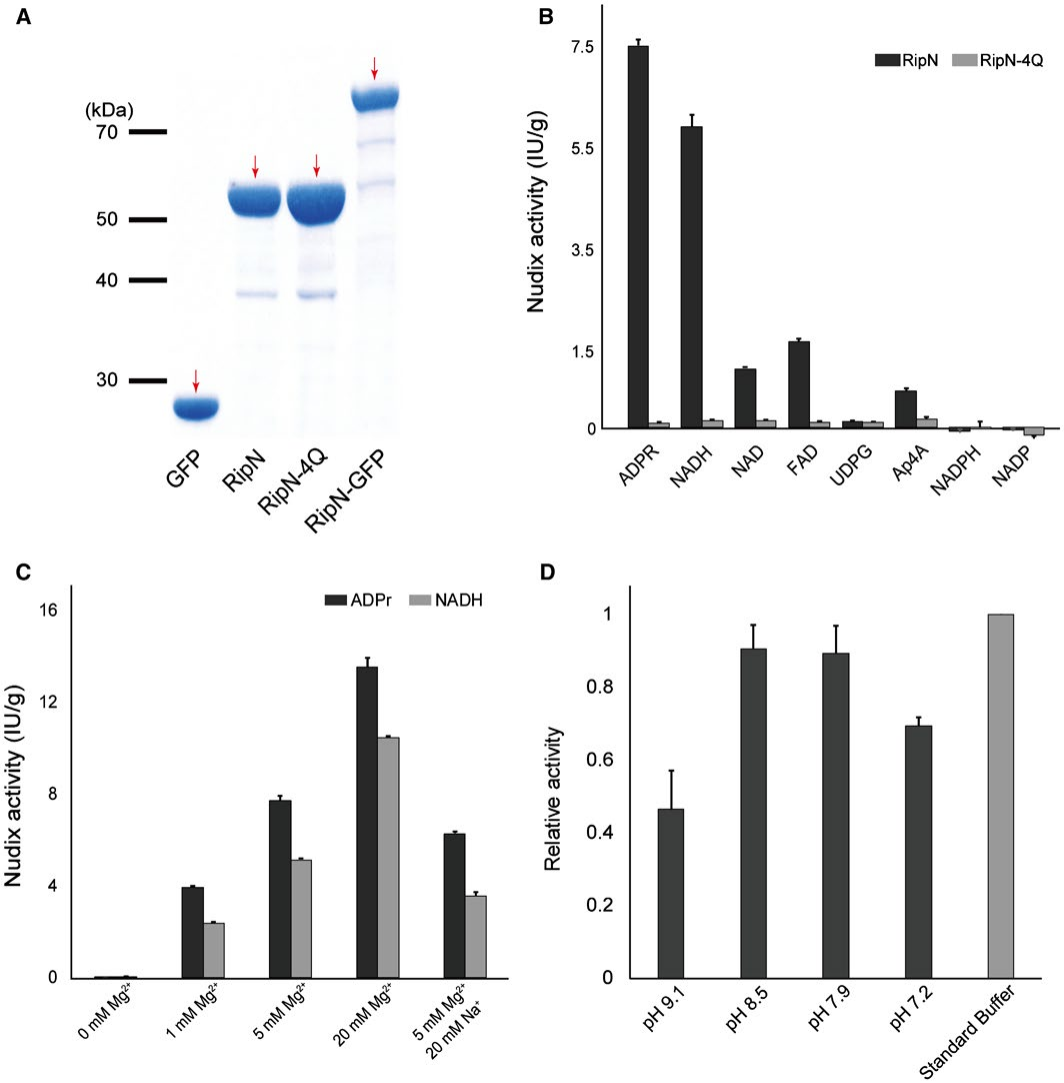
Heterogeneously expressed RipN, but not RipN‐4Q, shows ADP‐ribose (ADPr)/NADH pyrophosphorylase activity *in vitro*. (A) RipN, RipN‐4Q, green fluorescent protein (GFP) and RipN‐GFP were heterogeneously expressed with the *Escherichia coli* expression system and purified by Ni^2+^ affinity chromatography. Sodium dodecylsulfate‐polyacrylamide gel electrophoresis (SDS‐PAGE) was conducted for product analysis. Red arrows indicate the bands of the recombinant proteins. (B) Nudix hydrolase activity assays of RipN and RipN‐4Q *in vitro*. Different potential substrates and purified recombinant proteins of RipN and RipN‐4Q were added to the reaction systems at the same concentration. The Nudix hydrolase product was measured after 30 min of reaction. (C) Mg^2+^ is important for the Nudix hydrolase activity of RipN. The indicated concentrations of Mg^2+^ and Na^+^ were added to the reaction systems of the Nudix hydrolase activity assays, which used ADPr or NADH as the substrate. (D) The optimal pH for the Nudix hydrolase activity of RipN was pH 7.9–8.5. Different reaction systems with various pH values were tested using ADPr as the substrate. All experiments were biologically repeated three times or more with similar results.

Nudix hydrolases are found in all classes of organisms and hydrolyse a wide range of organic pyrophosphates with varying degrees of substrate specificity (Srouji *et al.*, 2017). RipN contains a Nudix box with conserved residues of the typical Nudix motif in its central region, which is hypothesized to hydrolyse a nucleoside diphosphate linked to some other moiety, X (Fig. 1B). In order to determine the enzymatic properties of RipN, we cloned the *ripN* gene from *R. solanacearum* GMI1000, and heterologously expressed and purified RipN and its mutant with an N‐terminal 6 × His tag (Fig. 2A).

Nudix enzymatic assays were performed using a series of substrates, referring to previous reports on Nudix hydrolases involved in plant immunity (Fig. 2B). The results showed that RipN can hydrolyse ADPr, NADH, NAD, FAD (flavin adenine dinucleotide) and Ap4A, with ADPr and NADH being the preferred substrates *in vitro *(Fig. 2B). However, RipN showed no activity in degrading uridine diphosphate (UDP)‐glucose or NADPH/NADP (reduced/oxidized forms of nicotinamide adenine dinucleotide phosphate) (Fig. 2B). The Nudix hydrolase activity of the non‐active mutant RipN‐4Q almost disappeared, compared with that of RipN, on ADPr, NADH, NAD, FAD or Ap4A. When using NADPH/NADP^+^ as the substrate, the value was lower than that of the blank control group. These results may be caused by the presence of monophosphate in NADPH/NADP^+^, which produces a high phosphate ion (Pi ion) background in the reaction system (Fig. S1B, see Supporting Information).

Previous studies have shown that Mg^2+^ occupancy of the catalytic site is indispensable for the activity of Nudix hydrolases (McLennan, 2006). In this study, we also found that Mg^2+^, but not Na^+^, was important for the Nudix hydrolase activity of RipN *in vitro* when using ADPr and NADH as substrates (Fig. 2C). In addition, we demonstrated that RipN has optimized *in vitro *activity between pH 7.9 and pH 8.5 when using ADPr as substrate (Fig. 2D). A fusion protein, RipN‐GFP (GFP, green fluorescent protein), was purified by the same method (Fig. 2A), and was confirmed to exhibit the same Nudix hydrolase activity as RipN (Fig. S5, see Supporting Information), indicating that RipN‐GFP can be used as an active Nudix hydrolase in further experiments. Taken together, these results demonstrate that RipN is a Nudix hydrolase whose preferred substrates are ADPr and NADH *in vitro*, and the conserved residues of the Nudix motif are indispensable for its hydrolase activity.

### RipN facilitates pathogen growth in *Arabidopsis* and suppresses PTI

**Figure 3:**
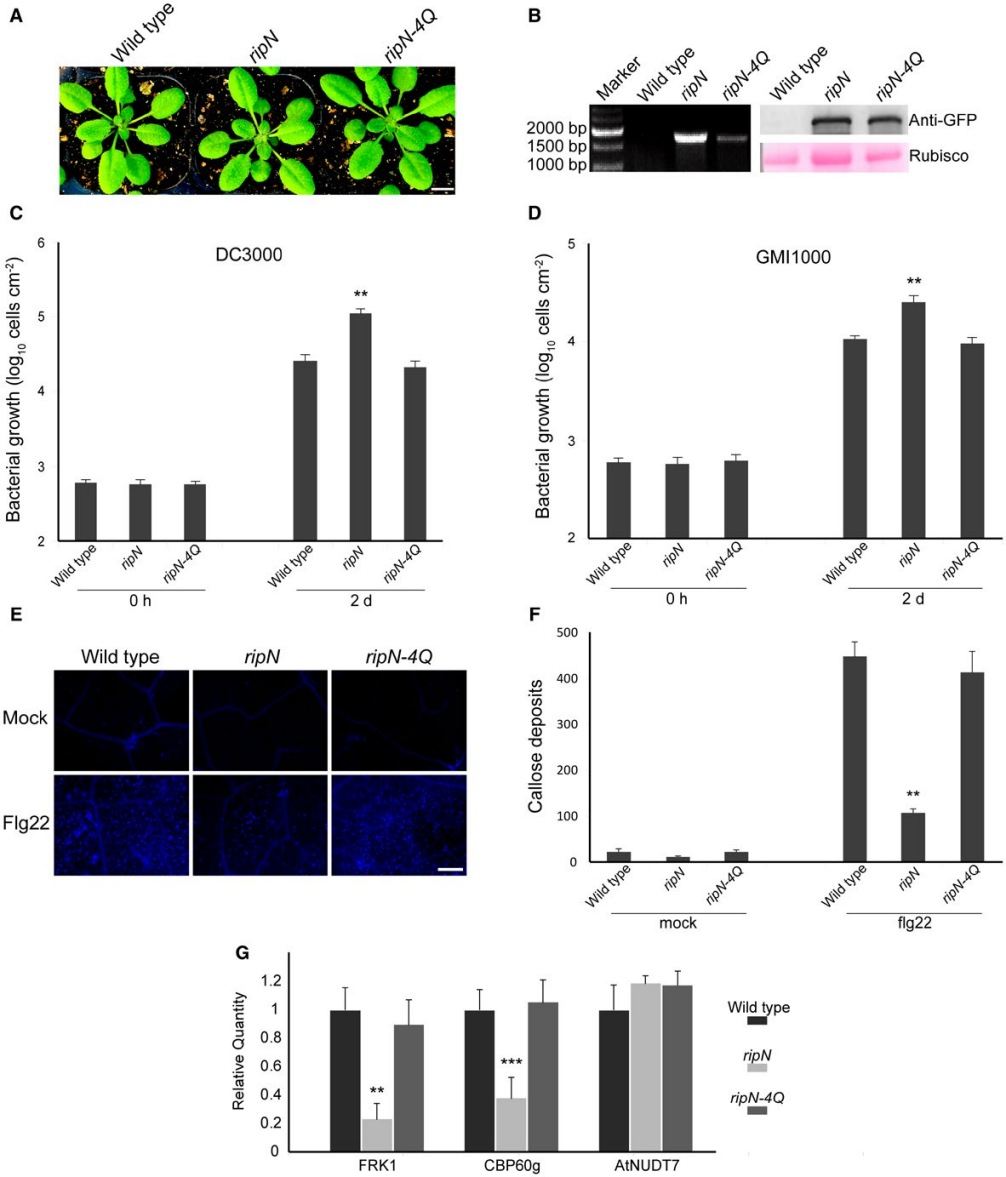
Expression of RipN with Nudix hydrolase activity suppresses the immunity of *Arabidopsis*. (A) Four‐week‐old plants expressing RipN or RipN‐4Q show no obvious morphological differences compared with wild‐type plants. (B) Reverse transcription‐polymerase chain reaction (RT‐PCR) and western blotting results show the transcription and expression of *ripN* and *ripN‐4Q* in transgenic Arabidopsis. Loading quantity was controlled by the Rubisco (ribulose‐1,5‐bisphosphate carboxylase/oxygenase, control) band depth using Ponceau S staining. (C, D) Bacterial population in Arabidopsis leaves. Five‐week‐old plant leaves of wild‐type, *ripN* transgenic and *ripN‐4Q* transgenic Arabidopsis were infiltrated with *Pseudomonas syringae* DC3000 and *Ralstonia solanacearum* GMI1000 bacteria at 2 × 10^5^ colony‐forming units (CFU)/mL. The leaf bacterial growth was determined at the indicated times after bacterial inoculation. Each data point consists of at least nine replicates (**P < 0.01, Student’s *t‐*test). (E, F) Diminished callose deposition in *ripN* transgenic plants. The indicated Arabidopsis lines were infiltrated with 1 mm MgCl_2_ or 1 μm flg22, and callose deposits were photographed at 10 h post‐infiltration. The figure shows representative images. Callose deposits were calculated using ImageJ software. Each data point represents the mean of four replicates (**P < 0.01, Student’s *t‐*test). (G) Transcriptional levels of the immune marker genes under flg22 treatment. Quantitative RT‐PCR was performed to analyse the expression levels of the indicated genes in 5‐week‐old leaves of wild‐type, *ripN* transgenic and *ripN‐4Q* transgenic Arabidopsis infiltrated with 1 μm flg22 at 7 h post‐infiltration. *AtACT1* was used as the reference gene. Each data point represents the mean of three replicates. Error bars indicate standard deviation (**P < 0.01, ***P < 0.001, Student’s *t‐*test).

To gain further information on the *in vivo* role of RipN, we constructed *ripN*‐ and *ripN‐4Q‐*overexpressing transgenic Arabidopsis (Fig. 3A). The transcription and expression of *ripN* and *ripN‐4Q* in transgenic Arabidopsis were confirmed by reverse transcription‐polymerase chain reaction (RT‐PCR) and western blot (Fig. 3B). Expression of RipN or RipN‐4Q does not affect the growth phenotype of Arabidopsis plants (Fig. 3A).

As a conserved effector within the *R. solanacearum* species complex, RipN is hypothesized to play a key role in interfering with host immunity *in vivo*. To test this, we first examined whether RipN could promote bacterial growth *in planta *by measuring the *in planta* growth of *R. solanacearum* GMI1000 and *Pseudomonas syringae *pv. *tomato* DC3000 strains in wild‐type (WT), *ripN *transgenic and *ripN‐4Q* transgenic Arabidopsis via leaf inoculation. Bacterial growth assays showed a three‐ to five‐fold higher growth rate of DC3000 and GMI1000 in *ripN* transgenic than in WT and *ripN‐4Q* transgenic Arabidopsis plants (Fig. 3C,D). This result indicates that RipN can facilitate the growth of pathogens *in planta*, and the activity of Nudix hydrolase is critical for the promotion of bacterial growth *in planta*.

To further investigate the role of RipN in the facilitation of pathogen growth in the host, we next evaluated PAMP‐induced callose deposition and mRNA accumulation of two PTI marker genes (*FRK1* and *CBP60g*) in WT, *ripN* transgenic and *ripN‐4Q* transgenic Arabidopsis plants treated with 1 μm of flg22. As shown in Fig. 3, there were no differences in flg22‐induced callose deposition and transcriptional levels of *FRK1* and *CBP60g* in WT plants and those expressing RipN‐4Q (Fig. 3E–G). However, callose deposition and mRNA levels of *FRK1* and *CBP60g *were significantly decreased in Arabidopsis expressing RipN (Fig. 3E–G). The mRNA levels of *AtNUDT7*, a Nudix hydrolase in *Arabidopsis*, were not affected by the expression of RipN or RipN‐4Q (Fig. 3G). In total, these data demonstrate that RipN with Nudix hydrolase activity promotes bacterial growth *in planta* by suppressing the PTI response in *Arabidopsis*.

### RipN localizes to the plant ER and nucleus

**Figure 4:**
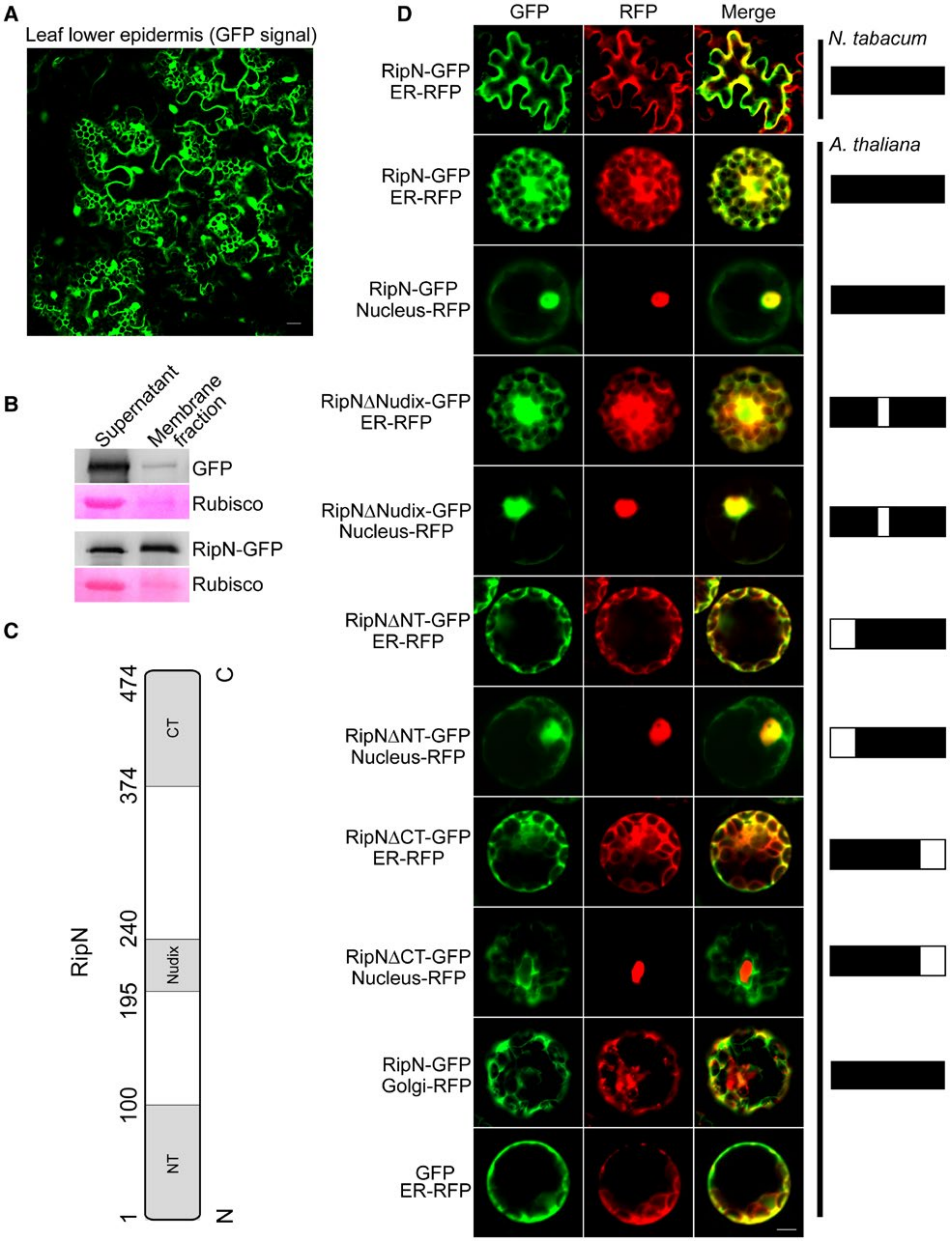
Subcellular localization of RipN and its mutations in plant cells. (A) Subcellular localization of RipN‐GFP in *ripN‐GFP *transgenic Arabidopsis leaves monitored by confocal laser scanning microscopy. GFP, green fluorescent protein. Bar, 10 μm. (B) The leaves of *GFP *and *ripN‐GFP *transgenic Arabidopsis were fully homogenized and ultracentrifuged without detergent. GFP, RipN‐GFP and Rubisco (ribulose‐1,5‐bisphosphate carboxylase/oxygenase) in the supernatant and membrane fraction components were detected by western blot or Ponceau S staining, respectively. (C) Schematic diagram of the RipN sequence. NT, N‐terminus; CT, C‐terminus. (D) Full‐length *ripN* or truncated *ripN* were cloned into pUC*35s‐GFP *and fused with *GFP*. The genes encoding proteins targeted to the endoplasmic reticulum (ER) (SALK_CD3‐959), nucleus (ARF4) or Golgi (SALK_CD3‐968) were cloned into pUC*35s‐RFP *to produce fusion proteins with red fluorescent protein (RFP). The plasmids were co‐transfected into Arabidopsis protoplasts, and fluorescence was detected after 15‐20 h by confocal microscopy. Representative photographs are shown. Bar, 10 μm. The solid box indicates the region of full‐length RipN, and the hollow box indicates the region of the truncated part of the corresponding RipN mutation.

Next, we conducted a fluorescence localization assay to investigate the cellular and molecular functionality of RipN. As shown in Fig. 4A, we observed a reticulate fluorescence pattern in the cytoplasm, accompanied by a strong nuclear signal, in the *ripN‐GFP *transgenic plant. These data suggest that RipN may localize to the plant cytoplasmic membrane system as well as the nucleus. To further test this, a membrane separation analysis was performed to identify both the soluble and membrane‐localized protein fractions from the cells of *GFP‐ *and *ripN‐GFP‐*expressing transgenic plants. As shown in Fig. 4B, using western blot analysis, RipN‐GFP was detected in the supernatant as well as within the membrane fraction, whereas almost all of the GFP and Rubisco (ribulose‐1,5‐bisphosphate carboxylase/oxygenase, control) were found within the supernatant fraction. These results show that RipN partly localizes to the plant cytoplasmic membrane system.

Next, we investigated whether RipN localizes to the nucleus and ER. To do this, co‐localization experiments were performed using known ER (SALK_CD3‐959), nucleus (ARF4) and Golgi (SALK_CD3‐968) markers. The full‐length or truncated *ripN *coding sequences were fused to GFP (Fig. 4C). For co‐localization in protoplasts, genes encoding the ER, nucleus or Golgi markers were cloned into pUC*35s‐*RFP to produce fusion proteins with red fluorescent protein (RFP). The resulting constructs were then co‐transfected into leaves of *N. tabacum* and Arabidopsis protoplasts. Using confocal microscopy, we observed that the full‐length RipN‐GFP, Nudix domain truncation and N‐terminal truncation were co‐localized with ER‐targeted RFP and nucleus‐targeted RFP (Fig. 4D). However, the C‐terminal truncation lost all of these localizations (Fig. 4D). This observation demonstrates that RipN‐GFP localizes to the ER and nucleus in *N. tabacum* leaf cells and Arabidopsis protoplasts, and the C‐terminus of RipN is essential for this localization *in planta*.

### RipN affects NADH/NAD^+^, but not GSH/GSSG (reduced/oxidized forms of glutathione) and ADP‐ribosylation, *in vivo*

**Figure 5:**
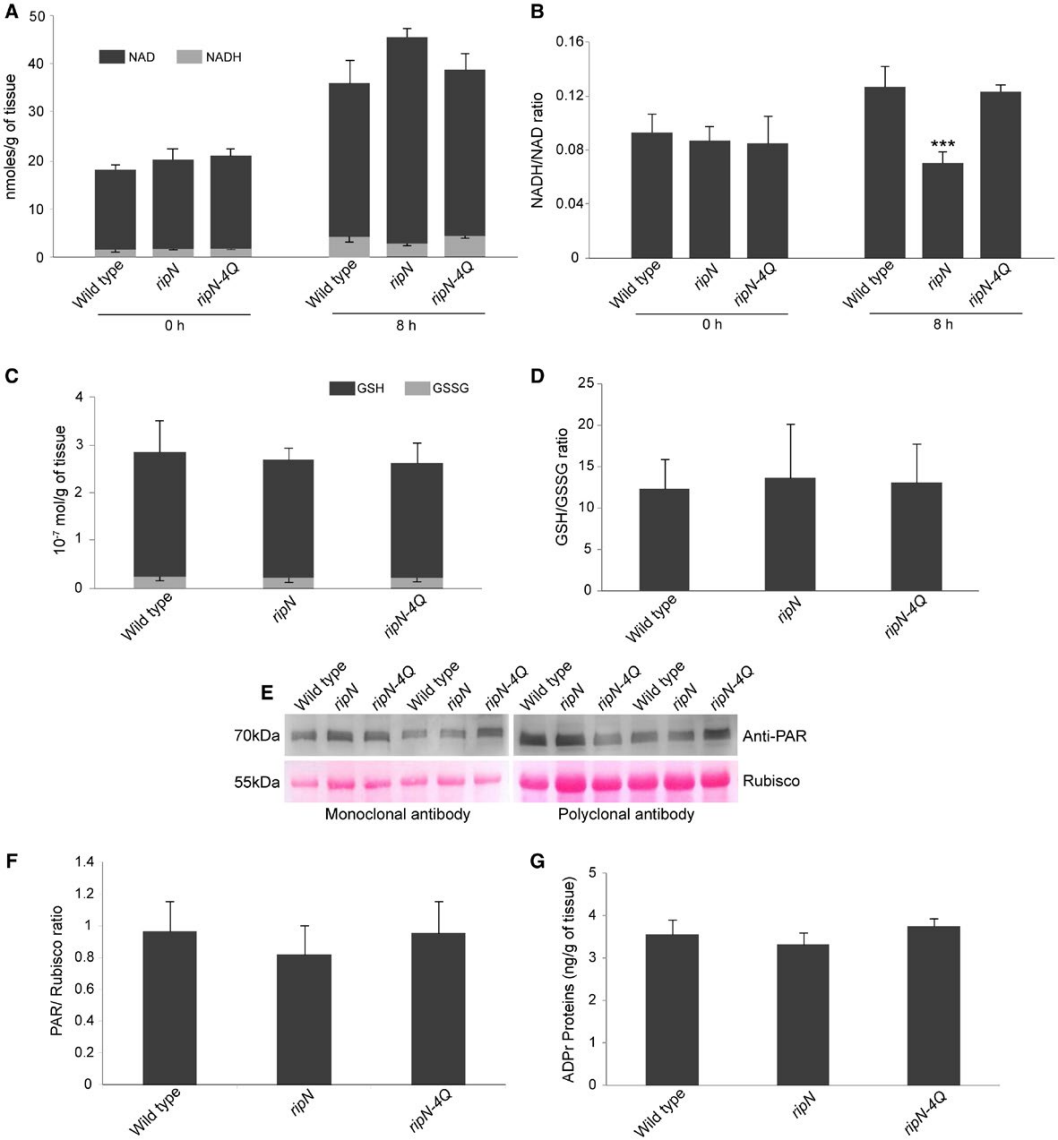
RipN alters the NADH/NAD^+^ ratio, but does not affect the GSH/GSSG ratio and ADP‐ribosylation, *in vivo. *(A, B) The levels of NADH and NAD^+^, and the NADH/NAD^+^ ratio, in pathogen‐challenged [infiltrated with 2 × 10^5^ colony‐forming units (CFU)/mL of *ripN* knockout strain GMI1000‐*ΔripN*] leaves of wild‐type, *ripN* transgenic and *ripN‐4Q* transgenic Arabidopsis plants at the indicated time points. Error bars represent the standard deviation derived from four biological experiments with three replicates in each experiment (***P < 0.001, Student’s *t‐*test). (C, D) The levels of GSH and GSSG, and the GSH/GSSG ratios, in pathogen‐challenged (infiltrated with 2 × 10^5^ CFU/mL of *ripN* knockout strain GMI1000‐*ΔripN*) leaves of wild‐type, *ripN* transgenic and *ripN‐4Q* transgenic Arabidopsis plants. Error bars represent the standard deviation derived from three biological experiments with three replicates in each experiment. (E) Detection of the amount of poly‐ADP‐ribosylated protein in wild‐type, *ripN* transgenic and *ripN‐4Q* transgenic Arabidopsis plants by monoclonal and polyclonal antibodies. The leaves of the indicated plants were treated with 1 μm flg22 at 8 h post‐infiltration (hpi). Rubisco was used as the loading control with staining by Ponceau S. (F) The relative amount of poly‐ADP‐ribosylated proteins in wild‐type, *ripN* transgenic and *ripN‐4Q* transgenic Arabidopsis plants determined by the control protein Rubisco. The quantification of ADP‐ribosylated proteins and Rubisco was performed using ImageJ. (G) Measurement of the quantity of ADP‐ribosylated proteins in wild‐type, *ripN* transgenic and *ripN‐4Q* transgenic Arabidopsis plants by enzyme‐linked immunosorbent assay (ELISA). Leaves of the indicated plants were treated with 1 μm flg22 at 8 hpi.

The results from the enzymatic assays above suggest that RipN may be a NADH/NAD^+^ modulator as it hydrolyses NADH (Fig. 2B). To test this hypothesis, we detected the levels of NADH/NAD^+ ^in the leaves of WT, *ripN* transgenic and *ripN‐4Q* transgenic Arabidopsis infected with a *ripN* knockout strain (GMI1000‐*ΔripN*) at 0 and 8 h post‐infection (hpi). As shown in Fig. 5A,B, there were no significant differences in the levels of NADH or NAD^+^ in WT, *ripN* transgenic and *ripN‐4Q* transgenic plants at 0 hpi. However, the expression of RipN caused a significantly lower ratio of NADH/NAD^+ ^at 8 hpi, compared with that in WT and *ripN‐4Q *transgenic plants. These results indicate that RipN may affect NADH/NAD^+^ indirectly *in planta *during immune activation and, furthermore, that its hydrolase activity is required for such biological function.

Given that RipN reduces the NADH/NAD^+^ ratio in infected host plants, we hypothesized that RipN may alter the redox homeostasis of plant cells during the early stages of pathogen‐induced resistance. To test this hypothesis, we measured the levels of GSH/GSSG in leaves of WT, *ripN* transgenic and *ripN‐4Q* transgenic Arabidopsis plants inoculated with GMI1000‐*ΔripN* using a GSH/GSSG assay kit. There was no significant difference in the amount of GSH or GSSG in these samples (5C,D).

In recent years, increasing evidence has suggested that ADP‐ribosylation participates in the activation of plant immunity (Feng *et al.*, 2015; Pham *et al.*, 2015; Song *et al.*, 2015). The fact that ADPr is one of the substrates of RipN in *in vitro* hydrolase activity assays (Fig. 2B) raises the possibility that proteins with ADPr or poly‐ADPr modification may be the physiological substrates of RipN. To test this hypothesis, we performed enzyme‐linked immunosorbent assay (ELISA) and western blotting assays to detect the amounts of ADP‐ribosylated and poly‐ADP‐ribosylated proteins in WT, *ripN* transgenic and *ripN‐4Q* transgenic Arabidopsis plants treated with the *ripN* knockout strain (GMI1000‐*ΔripN*) and flg22, respectively. In order to avoid the bias caused by the different types of antibody, we used both monoclonal and polyclonal antibodies of poly‐ADP‐ribosylation. As shown in 5E–G, there were no differences in the amounts of ADP‐ribosylated or poly‐ADP‐ribosylated proteins in flg22‐treated and GMI1000‐*ΔripN*‐infected plants at 8 hpi. These data demonstrate that ADPr and poly‐ADPr are not physiological substrates of RipN *in planta*, although ADPr is the preferred substrate of RipN *in vitro*.

In summary, the data above indicate that RipN affects NADH/NAD^+^, but not GSH/GSSG, ratios *in vivo* under infected conditions. Moreover, the activity of Nudix hydrolase is indispensable for these biochemical functions. In addition, although ADPr is the optimum substrate of RipN *in vitro*, RipN does not affect the amount of mono‐/poly‐ADP‐ribosylated proteins *in planta.*

## Discussion

Clarification of the biochemical functions of pathogen effector proteins not only improves our understanding of the underlying mechanisms of *R. solanacearum *pathogenesis, but also serves as a basis for the identification of potentially novel host immune signalling pathways. In recent years, the roles of Nudix hydrolase and NADH/NAD^+^ in plant innate immunity have attracted increased attention; however, the mechanism by which they affect immunity has not been fully elucidated (Fonseca and Dong, 2014; Ge *et al.*, 2007; Ishikawa *et al.*, 2010; Ogawa *et al.*, 2016; Pétriacq *et al.*, 2012, 2016; Yoshimura and Shigeoka, 2015). The present study demonstrates that RipN, a conserved T3E from *R. solanacearum*, has the typical ADPr/NADH pyrophosphorylase activity *in vitro*, localizes to the plant ER and nucleus, affects the NADH/NAD^+^ ratio of plant cells and inhibits the PTI of the host.

We detected the presence of *ripN* in all 30 fully genome sequenced *R. solanacearum* strains representative of the whole species diversity (Fig. 1A). This result demonstrates that RipN is extremely conserved in the *R. solanacearum* species complex, and also suggests that RipN plays a predominant, or indispensable, role in the infection process. In addition, phylogenetic analyses revealed that the diversification of RipN is closely related to geographical distribution (Fig. 1A). Historically, *Ralstonia* strains were subdivided into races based loosely on host range, or biovars based on their ability to oxidize various carbohydrates (Lowe‐Power *et al.*, 2018; Swanson *et al.*, 2005), but the current new DNA‐based classification system presents a more accurate vision into the different phylotypes (Ravelomanantsoa *et al.*, 2017; Yahiaoui *et al.*, 2017). This has led to the hypothesis that RipN is an ancient T3E which is indispensable for the full virulence of *R. solanacearum* and diverged with the *R. solanacearum* species complex when Pangea broke up 150–200 million years ago (Lowe‐Power *et al.*, 2018).

In this study, we have demonstrated that pathogenic bacteria grow more rapidly in *ripN* transgenic Arabidopsis plants than in WT plants (Fig. 3C,D), suggesting a role for RipN during infection. To further explore whether RipN is indispensable for the infection of *R. solanacearum*, we analysed the disease progress curves by root inoculation of the WT, the *ripN *deletion mutant and the *ripN* and *ripN‐4Q* complemented strains on Arabidopsis plants (Figs [Link], [Link] and [Link], [Link], see Supporting Information). No significant differences in virulence were observed before 8 days post‐infection, although the wilt symptoms appeared to be slightly delayed in plants inoculated with the *ripN *deletion mutant and *ripN‐4Q* complemented strain, compared with those infected by the WT and *ripN* complemented strain (Fig. S2). These results suggest that RipN may not be indispensable for the full virulence of *R. solanacearum* under these experimental conditions. However, given that RipN is a conserved T3E in the *R. solanacearum* species complex, which can cause severe bacterial wilt in more than 200 plant species (Swanson *et al.*, 2005), we hypothesize that RipN may exhibit a dominant virulence in specific hosts under diverse environmental factors, especially those mimicking the natural habitats in which the strains were isolated.

The Nudix protein superfamily comprises about 50 000 members (Pfam ID: CL0261) (Srouji *et al.*, 2017). However, only 171 have been characterized, and their physiological function and biochemical activity have not been conclusively demonstrated (Carreras‐Puigvert *et al.*, 2017; Srouji *et al.*, 2017; Yoshimura and Shigeoka, 2015). Although the Nudix effectors are regarded as a common weapon in the arsenal of plant pathogens (Dong and Wang, 2016), because of the wide differences in the sequences, structures and subcellular localizations of these Nudix effectors, their biological roles remain poorly understood (Carreras‐Puigvert *et al.*, 2017; Dong and Wang, 2016; Yoshimura and Shigeoka, 2015). To our knowledge, the biological functions of bacterial Nudix effectors have not been reported previously (Dong and Wang, 2016), and RipN is the first effector that shows typical ADPr/NADH pyrophosphorylase activity *in vitro*. Moreover, it is notable that the Nudix domain only occupies less than one‐tenth of the total length of RipN, suggesting that RipN may have additional unknown function *in planta*. In future studies, the identification of the target protein and the crystal structure of RipN will help us to further clarify the biological function of RipN.

Previous studies have directly indicated that ADP‐ribosylation facilitates the up‐regulation of plant immunity (Feng *et al.*, 2015; Pham *et al.*, 2015; Song *et al.*, 2015). The *in vitro* hydrolase activity assays described in this study raise the possibility that ADPr‐ or poly‐ADPr‐modified proteins may be the physiological substrates of RipN (Fig. 2B). However, we did not detect significant differences in the ADPr or poly‐ADPr level between WT, *ripN *and *ripN‐4Q *transgenic Arabidopsis plants treated with the *ripN* knockout strain (GMI1000‐*ΔripN*) and flg22 (5E–G). These results indicate that ADPr and poly‐ADPr are not likely to be physiological substrates of RipN. This notion is further supported by the result in a previous study of NUDT7, a Nudix protein in *Arabidopsis *(Ge *et al.*, 2007). A reasonable explanation is that *K*_cat_/*K*_m_ for the hydrolysis of ADPr by Nudix proteins is too high for ADPr to be an *in vivo* substrate (Ge *et al.*, 2007). In the present study, we detected a similar level of cellular redox homeostasis in untreated WT, *ripN* transgenic and *ripN‐4Q* transgenic Arabidopsis plants (5A,C). This is consistent with a previous study of NUDT7 (Ge *et al.*, 2007). However, it was reported that untreated *nudt7* plants accumulated over two‐fold more NADH than did WT plants (Jambunathan and Mahalingam, 2006). We found that there was a slight elevation of NAD^+^ in *ripN *transgenic Arabidopsis plants, and the NADH/NAD^+^ level in the leaves of *ripN *transgenic Arabidopsis plants at 8 hpi was approximately 50% lower than that in WT and *ripN‐4Q* transgenic plants (Fig. 5B). These results suggest that NADH, but not NAD^+^, could be a biologically important substrate of RipN, or that the enzyme activity of RipN to NAD^+^ is lower than that of RipN to NADH (Fig. 2B). However, we could not rule out the possibility that the lower NADH/NAD^+^ ratio in *ripN *transgenic Arabidopsis plants under infected conditions was indirectly generated by the Nudix‐catalysed degradation of an unknown key substrate that influences the equilibrium of NADH/NAD^+^.

GSH/GSSG is the principal redox buffer of the cell, and the ratio of GSH/GSSG is considered to be a major indicator of cellular redox status (Aon *et al.*, 2007; Gaucher *et al.*, 2018; Zhao *et al.*, 2009). Consistently, we also showed that the concentration of GSH was about 170‐fold higher than that of NADH in untreated and pathogen‐challenged tissues (Fig. 5A). These data may explain why the alteration in the ratio of NADH/NAD^+^ in *ripN *transgenic Arabidopsis did not affect the ratio of GSH/GSSG *in vivo *(5C,D), which suggests that RipN may not alter the cellular redox homeostasis on immune signalling. This hypothesis was further supported by the result of 3,3′‐diaminobenzidine (DAB) staining of the pathogen‐challenged leaves of WT, *ripN* transgenic and *ripN‐4Q* transgenic Arabidopsis plants (Fig. S4, see Supporting Information), where no obvious difference was detected. Taken together, these data suggest that NADH/NAD^+^ homeostasis can play an important role in plant immunity independent of its function as a sustainer of cellular redox homeostasis.

Although most proteins function in a single subcellular compartment, many are able to enter two or more compartments, a phenomenon known as dual or multiple targeting (Bauer *et al.*, 2013; Colcombet *et al.*, 2013; Gile *et al.*, 2015; Kalderon and Pines, 2014). In this study, we demonstrated that RipN is a dual targeting protein that locates at the ER and nucleus in plant cells (Fig. 4D). In most cases, the targeting signals are located at the N‐ or C‐terminus of proteins (Colcombet *et al.*, 2013; Gile *et al.*, 2015), and almost all of the secretion signals of *R. solanacearum* T3Es are located at the 50 N‐terminal residues of the proteins (Cunnac *et al.*, 2004). This may explain why the C‐terminus, but not the Nudix domain or N‐terminus of RipN, is indispensable for its localization (Fig. 4D). Interestingly, no signal for subcellular localization was identified in RipN via bioinformatics analysis, suggesting the existence of a non‐conventional localization signal within RipN. Alternatively, the specific localization of RipN could be associated with the interaction between RipN and its targets *in planta. *In this case, the C‐terminal truncation may destroy the three‐dimensional structure of RipN, leading to the disassociation of RipN and its target protein(s), and hence the failure of specific subcellular localization. The identification of the target protein(s) of RipN will further facilitate an understanding of the mechanism of localization and biological function of RipN in host cells.

## Experimental Procedures

### Plant materials and culture conditions

*Arabidopsis thaliana* Columbia‐0 ecotype (Col‐0) plants were grown in a growth room at 23 °C and 70% relative humidity with a 10‐h/14‐h day/night cycle for 4‐5 weeks before bacterial inoculation or protoplast isolation. Tobacco (*Nicotiana tabacum *cv. *xanthi*) plants were grown at 100/0 µmol/m^2^/s light radiance with a 16‐h/8‐h (24 °C/22 °C) light/dark cycle.

### Strains and plasmids

*Ralstonia solanacearum *GMI1000 WT and mutants were cultured in Casamino acid‐Peptone‐Glucose (CPG) medium at 30 °C, as described previously (Tasset *et al.*, 2010). The growth conditions, media and antibiotics used for the strains of *Escherichia coli* and *Agrobacterium tumefaciens* have been described previously (Lewis *et al.*, 2015). *Pseudomonas syringae *pv. *tomato* strain DC3000 was grown at 28 °C in King’s B medium (Lewis *et al.*, 2015). The *ripN* deletion strain (RSΔ*ripN*) was generated by performing pop‐in/pop‐out recombination with pK18mobsacB‐based plasmids, as described previously (Mukaihara *et al.*, 2016). To complement the *ripN *mutant, the *ripN* or *ripN‐4Q *(point mutations in four key residues for Nudix hydrolase activity: R219Q, E220Q, E223Q and E224Q) gene (including *ripN* native promoter) was cloned into pUC18T‐mini‐Tn7T‐Gm, and co‐transformed with pTNS2 to RSΔ*ripN* strain, generating the RSΔ*ripN*+*ripN *and RSΔ*ripN*+*ripN‐4Q* strains, respectively. To generate constructs for *A. thaliana* protoplast transfection, *E. coli* heterologous expression and *A. tumefaciens* infiltration assays, the *ripN* gene and derivatives were PCR amplified and inserted into pUC19‐*35S‐GFP*, pET28a+ and pBI121‐*GFP*, respectively. The primers used are listed in Table S1 (see Supporting Information).

### Generation of transgenic *A. thaliana* plants

To generate constructs for agroinfiltration transfection, *ripN *and *ripN‐4Q* genes were inserted into pBI121‐*GFP* (binary vector with a *GFP* gene as expression signal in frame with multiple cloning site (MCS), controlled by a cauliflower mosaic virus‐derived 35S promoter). Transformation of *A. thaliana* Col‐0 plants was performed using the floral dip method as described previously (Logemann *et al.*, 2006; Zhang *et al.*, 2018). Then, transformed plants were selected on Murashige–Skoog (MS) plates by western blot or fluorescence observation. From each T_0_ plant, seed (T_1_) was harvested; T_2_ plants were selected from kanamycin‐resistant T_1_ transformants. All T_2_ generation plants used for the experiment were analysed by western blot or fluorescence analysis.

### Bacterial inoculation

To analyse the *in planta* growth of GMI1000 and DC3000 strains in WT, *ripN* transgenic and *ripN‐4Q* transgenic Col‐0 plants, the GMI1000 and DC3000 cells were harvested and adjusted to 2 × 10^5^ colony‐forming units (CFU)/mL in 1 mm MgCl_2_. The suspensions were infiltrated into the leaves with a needleless syringe. Mock inoculation was performed with 1 mm MgCl_2_ without bacteria. Plants were kept at 90% humidity and bacterial numbers were derived as CFUs per square centimetre of infiltrated leaf tissue at 0 and 2 days post‐infiltration. *Agrobacterium*‐mediated transient expression in tobacco was performed as described previously (Yoo *et al.*, 2007). Before infiltration, the bacterial suspension was adjusted to a final optical density at 600 nm (OD_600 nm_) of 0.5. *Ralstonia solanacearum* root infection assays on *A. thaliana* plants were performed by soil‐soak inoculation, in which a bacterial suspension was poured over the soil of unwounded plants. To make inoculum, overnight‐grown bacterial suspension was diluted to obtain an inoculum of 3 × 10^8^ CFU/mL.

### Trypan blue and DAB staining

For trypan blue staining, leaves were sampled at 24 h post‐inoculation and transferred into trypan blue solution as described previously (Torres *et al.*, 2002). After boiling for 1 h, the leaves were destained for 24 h in chloral hydrate. DAB staining was carried out as described previously (Torres *et al.*, 2002) with minor modifications. Briefly, leaves at 24 h post‐inoculation were harvested and incubated in 1.0 mg/mL of DAB solution for 2 h in the dark, followed by boiling for 15 min in 90% ethanol.

### Callose deposition assay

Five‐week‐old Arabidopsis leaves were infiltrated with 1 μm flg22, collected 10 h later, stained with aniline blue and visualized with a fluorescence microscope, as described previously (Leslie *et al.*, 2016). Callose deposits were calculated using ImageJ software.

### Nudix activity assays

The recombinant proteins were expressed using *E. coli* BL21 (DE3) with pET28(a) containing the corresponding genes. Proteins isolated from *E. coli* were affinity purified following the manufacturer’s instructions (GE Healthcare Bio‐Sciences, Ni Sepharose™, Uppsala, Sweden). Nudix activity assays were performed as described previously (Ge *et al.*, 2007), with several modifications. For standard Nudix reaction buffer, NEBuffer 3 (NEB, B7003S, Beijing, China) diluted into 1× concentration was added with 0.4 µg/200 μL bovine alkaline phosphatase (NEB, M0290S). To test Nudix activity in different *in vitro* environments, 50 mm Tris‐HCl solutions with 1 mm DDT and various ions and pH values were prepared. The substrates ADPr (Sigma, A0752, Darmstadt, Germany), NADH (Sigma, N8129), NAD^+^ (Sigma, N6522), NADPH (Sigma, N5130), NADP^+^ (Sigma, N5755), FAD (Sigma, F6625), Ap4A (Sigma, D1262) and UDP‐glucose were dissolved in MilliQ water for 0.04 m preservation solution, stored at −80 ºC and diluted to 4 mm before use. A phosphate colorimetric kit (Sigma, MAK030) was used to measure Nudix activity.

### RNA isolation and quantitative RT‐PCR

Total RNA was extracted using an Eastep Total RNA Extraction Kit (Promega, Beijing, China) according to the manufacturer’s instructions, quantified on a Nanodrop spectrophotometer (Thermo Fisher Scientific, Waltham, Massachusetts, USA) and stored at −80 °C until use. For quantitative RT‐PCR, 800 ng of total RNA were used for first‐strand cDNA synthesis with a GoScript Reverse Transcription system (Promega). Next, quantitative RT‐PCR was performed using GoTaq qPCR Master Mix (Promega) on a Step One plus system (Applied Biosystems, Massachusetts, USA). The primers used for quantitative RT‐PCR are listed in Table S2 (see Supporting Information).

### NADH/NAD^+^ assay

NADH measurement was performed according to the manufacturer’s instructions (Abcam, ab65348, Cambridge, UK). To detect NADH, NAD^+^ needed to be decomposed before the reaction. To decompose NAD^+^, 200 mL of the extracted sample was aliquoted into 1.5‐mL Eppendorf tubes and heated to 60 °C for 30 min. The optical density of the denatured lysates was measured at 450 nm. NADH in the sample wells was calculated according to the NADH standard curve.

### Measurement of plant GSH/GSSG and poly(ADP‐ribose) (PAR)

Total protein of 4‐week‐old Col‐0 was extracted from 0.2 g of tissue by grinding with 200 μL of extraction buffer (50 mm phosphate buffer, pH 7.0, 1% Triton X‐100). Plant GSH/GSSG was measured by a GSH/GSSG Test Kit (Beyotime, #S0053, Shanghai, China). For the detection of PAR proteins with western blot, mouse anti‐poly(ADPr) (PAR) (USB‐045159) and mouse anti‐PolyAR (Trevigen‐4335‐MC‐100‐ac) were used.

### Accession numbers

The protein and DNA sequences described in this article have been deposited at GenBank under the accession numbers CAD18281.1 and AL646053.1. Details of the DNA primers are given in Table S1.

## Conflicts of Interest

The authors have no conflicts of interest to declare.

## Supporting information


**Fig. S1  **General principle of Nudix hydrolase activity measurement. Recombinant proteins (Nudix hydrolase) isolated from *E. coli* were purified by His‐tag affinity chromatography. For standard Nudix reactions, Buffer 3 (NEB, B7003S) diluted into 1×concentration was added with 0.4 ug/200 μL bovine alkaline phosphatase (AP, NEB, M0290S). For substrates, ADPr (Sigma, A0752), NADH (Sigma, N8129), NAD^+^ (Sigma, N6522), NADPH (Sigma, N5130), NADP^+^ (Sigma, N5755), FAD (Sigma, F6625), Ap4A (Sigma, D1262), and UDP‐glucose were solved in MilliQ water for 0.04 M preservation solution and stored at ‐80℃ and diluted to 4 mM before usage. Phosphate colorimetric Kit (Sigma, MAK030) is used to measure the produced phosphate as the standard as Nudix activity. (A) Substrates without monophosphate (e.g., NADH/NAD^+^). (B) Substrates with monophosphate (e.g., NADPH/NADP^+^).

**Fig. S2  **Disease progress curves of *R*. *solanacearum *GMI1000 wild‐type strain, *ripN *deletion strain and the *ripN* and *ripN‐4Q* complement strains on Arabidopsis plants. Average disease indices following root inoculation of 16 Arabidopsis plants per treatment by bacterial suspensions containing 3×10^8^ CFU mL^‐1 ^of the wild type strain GMI1000, *ripN *deletion mutant strain and* ripN *and *ripN‐4Q* complement strains. Wilting symptoms were recorded over time according to a disease index scale (0: no wilting, 1: 25% wilted leaves, 2: 50%, 3: 75%, 4: death). Error bars indicate standard errors.

**Fig. S3  **Construction of *ripN* knockout strain and the *ripN* and *ripN‐4Q* complement strains. (A) PCR analysis of positive knockout strains (*ripN‐1* and *ripN‐2*). The positive strains produce a prospective band of 616 bp length (red arrow). (B) Secretion of the complement proteins. Complemented strains were cultivated overnight at 30°C in the CPG medium, then cells were harvested and resuspended in M63 glucose minimal medium (Arlat* et al.*, 1994) (containing 100 μg of Congo red per milliliter) at OD600=0.5. After 16 h incubation, the culture was centrifuged at 1500×g for 30 min, and then the culture supernatant was filtered with 0.2 μm pore filter. The culture supernatant was concentrated to 200‐250 folds by ultra‐filtration. The western blotting was performed with HA‐tag antibody.

**Fig. S4  **Detection of plant cell death and ROS accumulation. (A). Detection of plant cell death and ROS accumulation by trypan blue staining and DAB staining. Indicated Arabidopsis leaves were infiltrated with *Pst *DC3000‐AvrRpm1 of 10^8^ CFUmL^−1^. Photographs were taken at 24 hr postinfiltration. (B) The levels of plant cell death and ROS accumulation in the indicated leaves were calculated using ImageJ software. Each data point represents the mean of six replicates.

**Fig. S5  **Heterogenous expressed RipN‐GFP, but not RipN‐4Q‐GFP, has the ADP‐ribose/NADH pyrophosphorylase activity *in vitro*. (A) RipN‐GFP and RipN‐4Q‐GFP were heterogenous expressed with *E.coli* expression system and purified by Ni^2+ ^affinity chromatography. Western blot analysis of expression of indicated proteins. The red arrows indicate the bands of the recombinant proteins. (B) Nudix hydrolase activity assays of RipN‐GFP and RipN‐4Q‐GFP *in vitro*. Different substrates which might be degraded by Nudix hydrolase, and purified recombinant proteins of RipN‐GFP and RipN‐4Q‐GFP were added into reaction systems with the same concentration. Nudix hydrolase activities were determined after 30 minutes of reaction. All experiments were biologically repeated three times with similar results.

**Table S1  **DNA primers used in *ripN* knockout and *ripN *complementation assays.

**Table S2  **DNA primers used for qRT‐PCR.
