# Supplementary figures and images for: The Ralstonia solanacearum effector RipN suppresses plant PAMP‐triggered immunity, localizes to the endoplasmic reticulum and nucleus, and alters the NADH/NAD+ ratio in Arabidopsis
Source: Mol Plant Pathol. 2019 Feb 18;20(4):533–46. doi: 10.1111/mpp.12773 (PMC6637912; doi:10.1111/mpp.12773)

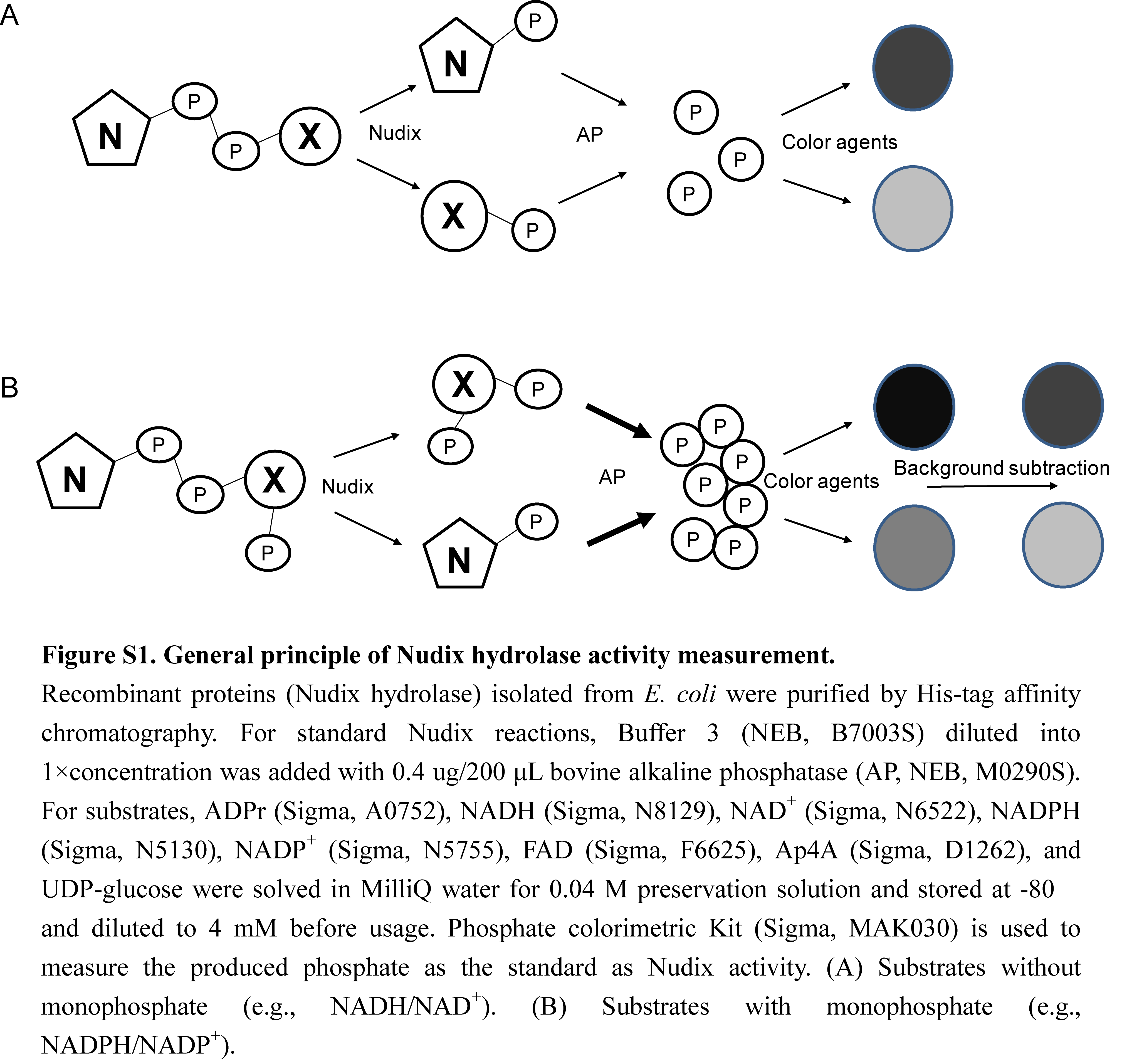

Supplement: Supplementary file 1 — Fig. S1 General principle of Nudix hydrolase activity measurement. Recombinant proteins (Nudix hydrolase) isolated from E. coli were purified by His‐tag affinity chromatography. For standard Nudix reactions, Buffer 3 (NEB, B7003S) diluted into 1×concentration was added with 0.4 ug/200 μL bovine alkaline phosphatase (AP, NEB, M0290S). For substrates, ADPr (Sigma, A0752), NADH (Sigma, N8129), NAD+ (Sigma, N6522), NADPH (Sigma, N5130), NADP+ (Sigma, N5755), FAD (Sigma, F6625), Ap4A (Sigma, D1262), and UDP‐glucose were solved in MilliQ water for 0.04 M preservation solution and stored at ‐80℃ and diluted to 4 mM before usage. Phosphate colorimetric Kit (Sigma, MAK030) is used to measure the produced phosphate as the standard as Nudix activity. (A) Substrates without monophosphate (e.g., NADH/NAD+). (B) Substrates with monophosphate (e.g., NADPH/NADP+). [file MPP-20-533-s001.tif]

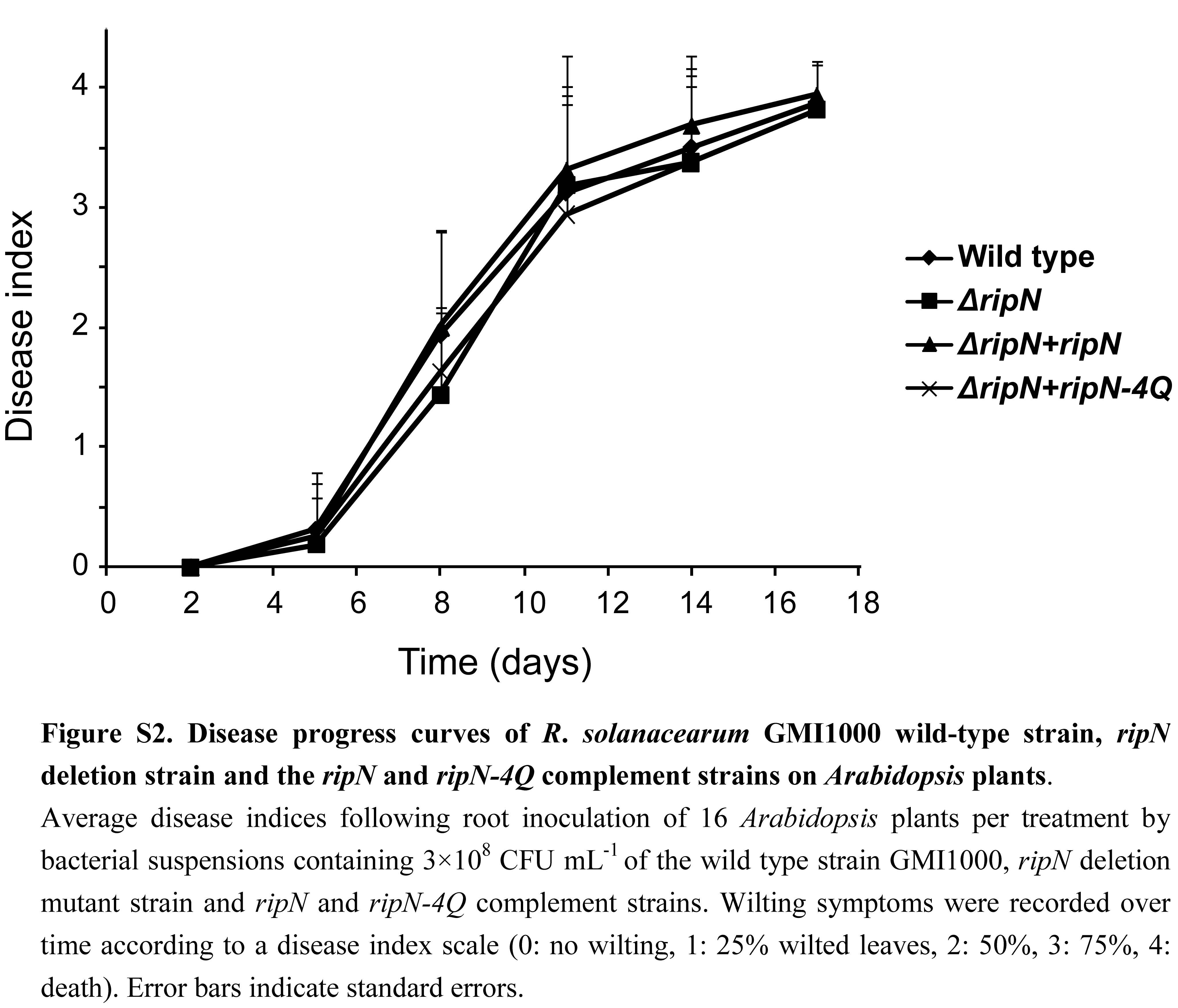

Supplement: Supplementary file 2 — Fig. S2 Disease progress curves of R. solanacearum GMI1000 wild‐type strain, ripN deletion strain and the ripN and ripN‐4Q complement strains on Arabidopsis plants. Average disease indices following root inoculation of 16 Arabidopsis plants per treatment by bacterial suspensions containing 3×10^8^ CFU mL‐1 of the wild type strain GMI1000, ripN deletion mutant strain and ripN and ripN‐4Q complement strains. Wilting symptoms were recorded over time according to a disease index scale (0: no wilting, 1: 25% wilted leaves, 2: 50%, 3: 75%, 4: death). Error bars indicate standard errors. [file MPP-20-533-s002.tif]

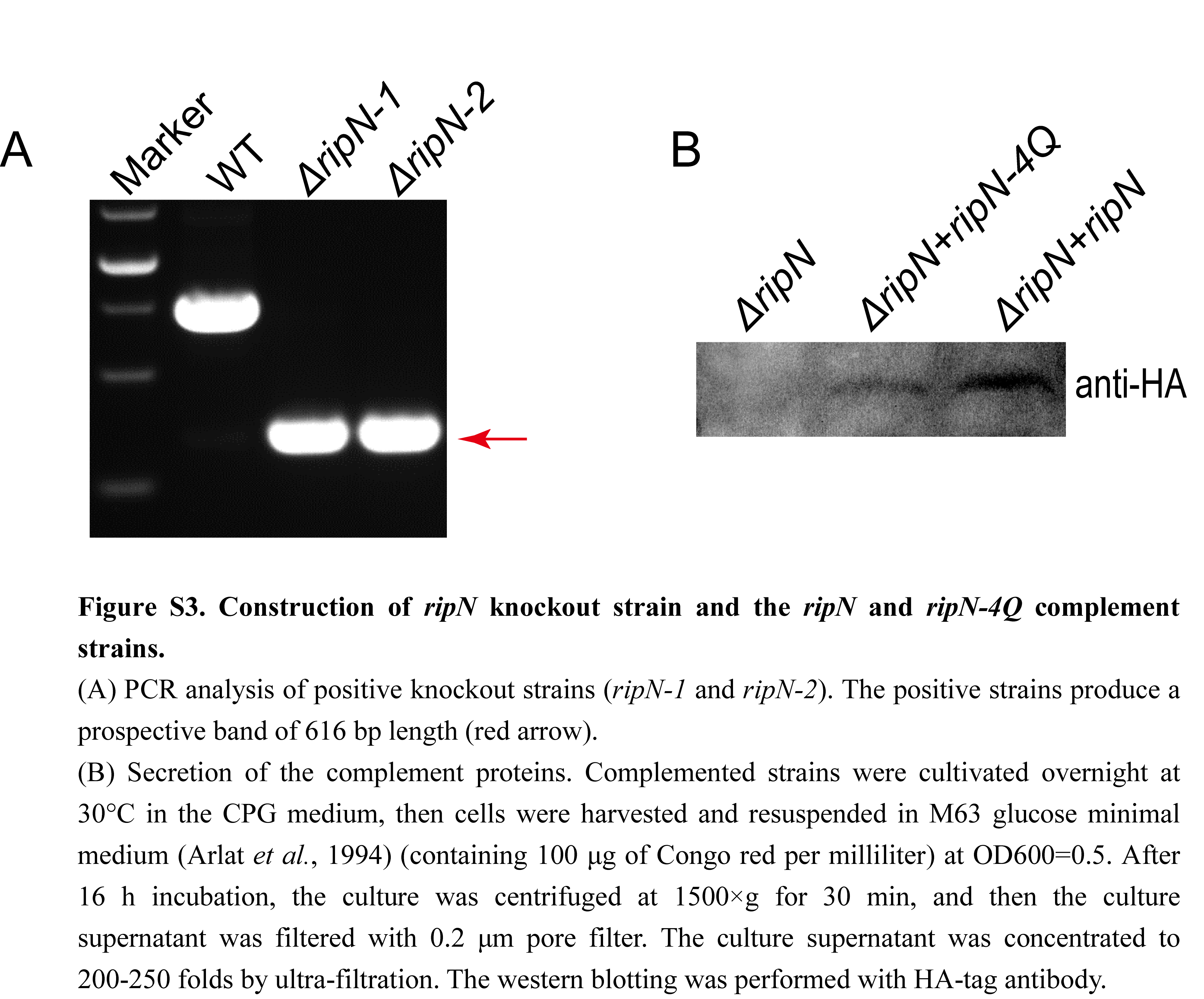

Supplement: Supplementary file 3 — Fig. S3 Construction of ripN knockout strain and the ripN and ripN‐4Q complement strains. (A) PCR analysis of positive knockout strains (ripN‐1 and ripN‐2). The positive strains produce a prospective band of 616 bp length (red arrow). (B) Secretion of the complement proteins. Complemented strains were cultivated overnight at 30°C in the CPG medium, then cells were harvested and resuspended in M63 glucose minimal medium (Arlat et al., 1994) (containing 100 μg of Congo red per milliliter) at OD600=0.5. After 16 h incubation, the culture was centrifuged at 1500×g for 30 min, and then the culture supernatant was filtered with 0.2 μm pore filter. The culture supernatant was concentrated to 200‐250 folds by ultra‐filtration. The western blotting was performed with HA‐tag antibody. [file MPP-20-533-s003.tif]

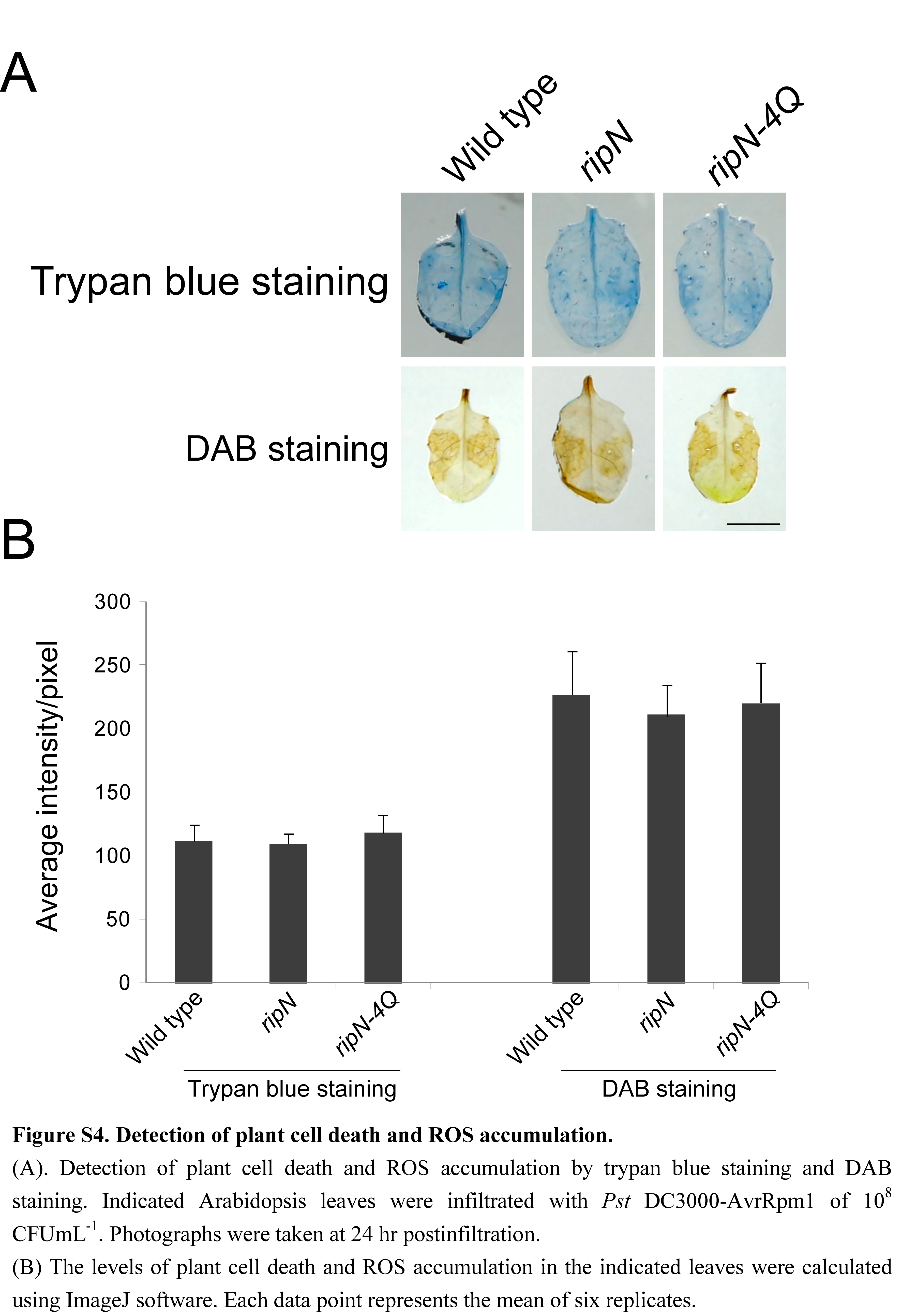

Supplement: Supplementary file 4 — Fig. S4 Detection of plant cell death and ROS accumulation. (A). Detection of plant cell death and ROS accumulation by trypan blue staining and DAB staining. Indicated Arabidopsis leaves were infiltrated with Pst DC3000‐AvrRpm1 of 108 CFUmL−1. Photographs were taken at 24 hr postinfiltration. (B) The levels of plant cell death and ROS accumulation in the indicated leaves were calculated using ImageJ software. Each data point represents the mean of six replicates. [file MPP-20-533-s004.tif]

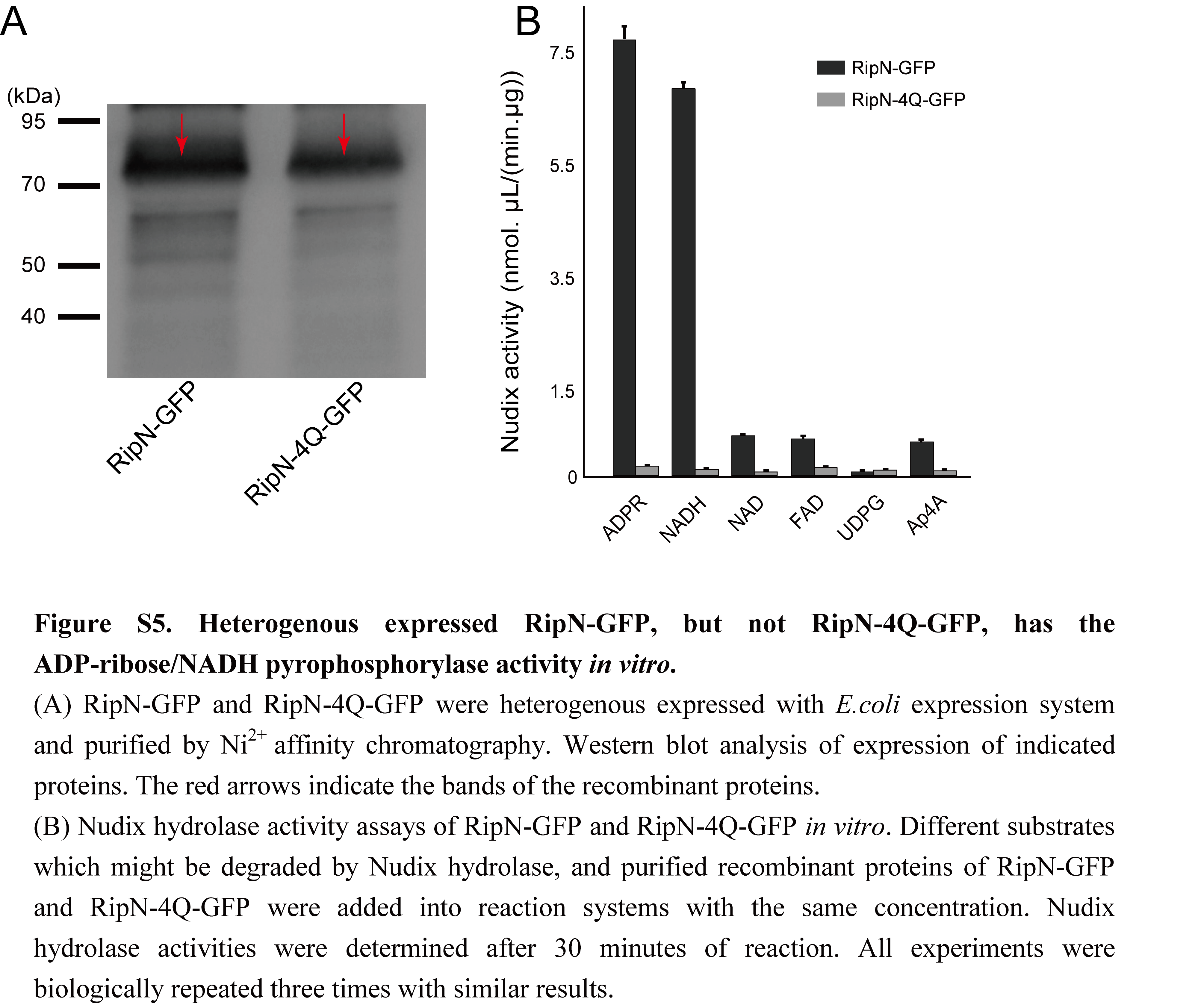

Supplement: Supplementary file 5 — Fig. S5 Heterogenous expressed RipN‐GFP, but not RipN‐4Q‐GFP, has the ADP‐ribose/NADH pyrophosphorylase activity in vitro. (A) RipN‐GFP and RipN‐4Q‐GFP were heterogenous expressed with E.coli expression system and purified by Ni2+ affinity chromatography. Western blot analysis of expression of indicated proteins. The red arrows indicate the bands of the recombinant proteins. (B) Nudix hydrolase activity assays of RipN‐GFP and RipN‐4Q‐GFP in vitro. Different substrates which might be degraded by Nudix hydrolase, and purified recombinant proteins of RipN‐GFP and RipN‐4Q‐GFP were added into reaction systems with the same concentration. Nudix hydrolase activities were determined after 30 minutes of reaction. All experiments were biologically repeated three times with similar results. [file MPP-20-533-s005.tif]
