# Supplementary material for: The Ralstonia solanacearum effector RipN suppresses plant PAMP‐triggered immunity, localizes to the endoplasmic reticulum and nucleus, and alters the NADH/NAD+ ratio in Arabidopsis
Source: Mol Plant Pathol. 2019 Feb 18;20(4):533–46. doi: 10.1111/mpp.12773 (PMC6637912; doi:10.1111/mpp.12773)
Supplement: Supplementary file 6 — Table S1 DNA primers used in ripN knockout and ripN complementation assays. [file MPP-20-533-s006.docx]

**Table S1.** DNA primers used in *ripN* knockout and *ripN* complementation assays.

| Target gene | Comment for product | Forward primer 5’-3’ | Reverse primer 5’-3’ |
| --- | --- | --- | --- |
| *ripN*-upstream | introducing Δ*ripN* | ccggaattcTCGGTCATCGCGTTCAGTTCTG | GTCGTGAGGGTTCCGGGTTG |
| *ripN*-downstream | introducing Δ*ripN* | ACGGCGAGCGAGACCTCGGG | gctctagaCAATTGGTATTCGATAGAG |
| +*ripN* | *ripN* complement | cgcggatccCTCAGACGCAGGCTGTGAGC | ccgctcgagAGCAGCCTGTCGGATCGCCG |
| *ΔripAK-test* | Knockout test | ATCAAGAACCGCTTCCCCTA | AGGCATAGCATCCGTTGTCC |

^a^The restriction sites used for cloning are underlined.
