# Supplementary material for: The Ralstonia solanacearum effector RipN suppresses plant PAMP‐triggered immunity, localizes to the endoplasmic reticulum and nucleus, and alters the NADH/NAD+ ratio in Arabidopsis
Source: Mol Plant Pathol. 2019 Feb 18;20(4):533–46. doi: 10.1111/mpp.12773 (PMC6637912; doi:10.1111/mpp.12773)
Supplement: Supplementary file 7 — Table S2 DNA primers used for qRT‐PCR. [file MPP-20-533-s007.docx]

**Table S2.** DNA primers used for qRT-PCR

| Protein Name | Forward primer 5’-3’ | Reverse primer 5’-3’ |
| --- | --- | --- |
| Actin (ACT1) | TCTTGATCTTGCTGGTCGTG | GAGCTGGTTTTGGCTGTCTC |
| FRK1 | GCCAACGGAGACATTAGAG | CCATAACGACCTGACTCATC |
| CBP60g | AAGAAGAATTGTCCGAGAGGAG | GGCGAGTTTATGAAGCACAG |
| ATNUDX7 | AAGTCTTAGGGCTTCTCTTTCGC | GGGATTGTATCAGGAGTTTCAGAGA |
